# Use of psychotropic medications during pregnancy and the postpartum period: Review on Recent Works and Clinical Scenarios

**DOI:** 10.1192/j.eurpsy.2023.2393

**Published:** 2023-07-19

**Authors:** E. Román, M. Natividad, J. Cobo, R. Ayesa, H. Cachinero, I. Figueras, E. Izquierdo, E. Martínez, J. P. Paolini San Miguel, J. A. Monreal, A. González-Rodríguez

**Affiliations:** 1 Mental Health, Mutua Terrassa University Hospital. University of Barcelona (UB). CIBERSAM; 2 Mental Health, Mutua Terrassa University Hospital. Fundació Docència i Recerca. University of Barcelona. CIBERSAM., Terrassa; 3 Mental Health, Parc Tauli University Hospital. Autonomous University of Barcelona (UAB). I3PT. CIBERSAM, Sabadell; 4 Psychiatry, Marqués Valdecilla University Hospital. IDIVAL. National University of Distance Education. CIBERSAM, Santander; 5 Mental Health, Mutua Terrassa University Hospital. University of Barcelona. Fundació Docència i Recerca Mutua Terrassa; 6 Mental Health, Mutua Terrassa University Hospital. Fundació Docència i Recerca Mutua Terrassa. University of Barcelona; 7 Mental Health, Mutua Terrassa University Hospital. Fundació Docència i Recerca Mutua Terrassa. University of Barcelona., Terrassa; 8 Mental Health, Parc Tauli University Hospital. Autonomous University Hospital. I3PT. CIBERSAM, Sabadell; 9 Mental Health, Mutua Terrassa University Hospital. Fundació Docència i Recerca Mutua Terrassa. University of Barcelona. CIBERSAM. Inst. Neurociències UAB; 10 Mental Health, Mutua Terrassa University Hospital. Fundació Docència i Recerca Mutua Terrassa. University of Barcelona. CIBERSAM, Terrassa, Spain

## Abstract

**Introduction:**

The effects of antidepressant and antipsychotic medications in the perinatal period in both mothers and children have been a subject of interest for many decades. Risks and benefits should be considered according to the illness stage, trimester of pregnancy/ postpartum period, and neonatal outcomes.

**Objectives:**

Our goal was to summarize the knowledge about the use of antidepressants and antipsychotics in the perinatal period. To illustrate the complexity of treatment decisions with clinical reports.

**Methods:**

Review: A narrative review was carried out using the PubMed database including papers published in 2022. Evidence about the risks and benefits of using antidepressants and antipsychotics in the perinatal period is presented. Search terms: antidepressants OR antipsychotics AND (perinatal OR pregnancy OR postpartum).

Case reports (5 clinical scenarios): (1) pre-pregnancy counselling, (2-4) first-, second- and third-trimester of pregnancy, and (5)postpartum/breastfeeding.

**Results:**

Review: (1)Depression/antidepressants. Treating maternal depressive symptoms is associated with a lower risk of pregnancy complications. Although placental passage of sertraline is low, drug monitoring is recommended. Antidepressant use in pregnancy is associated with preterm delivery and low weight at birth. (2)Psychosis/Antipsychotics. Antipsychotic intrauterine exposure is not significantly associated with increased risk of major congenital malformations. Minimum effective doses are recommended.

Case reports. (1)Pre-pregnancy counselling. Schizoaffective disorder receiving perphenazine, quetiapine and lithium. (2)First-trimester pregnancy. Discontinuation of treatment in major depressive disorder. (3-4)Second/third trimester. Occurrence of anxiety symptoms in posttraumatic stress disorder. (5)Postpartum/Breastfeeding. Discontinuation of antidepressants.

**Conclusions:**

Shared decision-making models for antidepressants and antipsychotics prescription represent patient-centered approaches to be recommended in perinatal period.

**Disclosure of Interest:**

None Declared

